# Isolation and characterization of multipotent mesenchymal stromal cells from the gingiva and the periodontal ligament of the horse

**DOI:** 10.1186/1746-6148-7-42

**Published:** 2011-08-02

**Authors:** Niels Mensing, Hagen Gasse, Nina Hambruch, Jan-Dirk Haeger, Christiane Pfarrer, Carsten Staszyk

**Affiliations:** 1 Institute of Anatomy, University of Veterinary Medicine Hannover, Bischofsholer Damm 15, D-30173 Hannover, Germany

## Abstract

**Background:**

The equine periodontium provides tooth support and lifelong tooth eruption on a remarkable scale. These functions require continuous tissue remodeling. It is assumed that multipotent mesenchymal stromal cells (MSC) reside in the periodontal ligament (PDL) and play a crucial role in regulating physiological periodontal tissue regeneration. The aim of this study was to isolate and characterize equine periodontal MSC.

Tissue samples were obtained from four healthy horses. Primary cell populations were har-vested and cultured from the gingiva, from three horizontal levels of the PDL (apical, midtooth and subgingival) and for comparison purposes from the subcutis (masseteric region). Colony-forming cells were grown on uncoated culture dishes and typical *in vitro *characteristics of non-human MSC, i.e. self-renewal capacity, population doubling time, expression of stemness markers and trilineage differentiation were analyzed.

**Results:**

Colony-forming cell populations from all locations showed expression of the stemness markers CD90 and CD105. In vitro self-renewal capacity was demonstrated by colony-forming unit fibroblast (CFU-F) assays. CFU-efficiency was highest in cell populations from the apical and from the mid-tooth PDL. Population doubling time was highest in subcutaneous cells. All investigated cell populations possessed trilineage differentiation potential into osteogenic, adipogenic and chondrogenic lineages.

**Conclusions:**

Due to the demonstrated in vitro characteristics cells were referred to as equine subcutaneous MSC (eSc-MSC), equine gingival MSC (eG-MSC) and equine periodontal MSC (eP-MSC). According to different PDL levels, eP-MSC were further specified as eP-MSC from the apical PDL (eP-MSCap), eP-MSC from the mid-tooth PDL (eP-MSCm) and eP-MSC from the subgingival PDL (eP-MSCsg). Considering current concepts of cell-based regenerative therapies in horses, eP-MSC might be promising candidates for future clinical applications in equine orthopedic and periodontal diseases.

## Background

The periodontium represents the supporting apparatus of the tooth. It is composed of four constituents: the dental cementum, the alveolar bone, the gingiva and the periodontal ligament (PDL). The PDL is a highly cellular and vascular connective tissue which fills the periodontal space between the dental cementum and the alveolar bone. In occlusal direction the PDL is continuous with the connective tissue of the gingiva. The collagen fiber apparatus of the PDL is well adapted to anchor the tooth in the jaw [1,2]. During mastication, tendon-like collagen bundles of the PDL are capable of withstanding displacing forces and thus protecting the tooth from mechanical damage [3-5].

A unique feature of the PDL is an exceptional high rate of remodeling which is reflected by a very rapid collagen turnover [6-8]. It has not been fully understood whether this feature is a consequence of steady masticatory loads or if it is an inherent property of the PDL [6]. However, tissue remodeling and collagen turnover are essential prerequisites for several functional characteristics of the PDL. Under physiological conditions the PDL needs to be adjusted continuously in response to normal tooth drift and tooth eruption [1,9]. Moreover, continuous repairs and replacements of exhausted matrix components are urgently needed as the PDL is subjected to a variety of mechanical loads during mastication [1]. Under pathological conditions, periodontal remodeling facilitates the healing and functional regeneration of injured tissue areas [10-12]. The regulation and control of periodontal remodeling and homeostasis have been the subject of several studies proposing a key role of the cellular fraction of the PDL [13,14]. Special attention has been paid to the question whether the different formative cell types of the periodontium (cementoblasts, PDL-fibroblasts, osteoblasts) arise from a common precursor or if specific precursor cells exist for each of the cell types [3,15]. Meanwhile, studies have demonstrated the existence of distinct cells within the PDL; which have been termed periodontal ligament stem cells (PLSC) [16,17]. PLSC possess the capacity of multilineage differentiation in vitro and have recently been identified in the PDL of men, e.g. [18-20], rats [21] and sheep [14]. In vivo, PLSC are thought to be the progenitors of the formative cells of the periodontium (e.g. cementoblasts, PDL-fibroblasts and osteoblasts) which in turn are required to enable continuous periodontal remodeling and regeneration [16,22].

It has been proposed that PLSC can be utilized as a cell source for the treatment of periodon-tal diseases, i.e. for new concepts in tissue engineering and for stem cell-based regenerative therapies [23,24]. In this regard, the equine periodontium appears to possess capacities for tissue regeneration and tissue remodeling exceeding those of other species by far. Support for this assumption can be derived from the unique dental and periodontal anatomy of the horse [2,25]. The equine PDL and gingiva are challenged in a very particular way. The highly abrasive diet causes a massive tooth wear rate of approx. 3 to 4 mm per year, with an extreme wear rate of up to 9 mm per year [26]. The occlusal loss of equine dental substances is compensated by a continuous eruptive movement of the tooth at an adequate rate. In comparison, brachyodont teeth of man move only between 0.02 and 0.3 mm per year under physiological conditions [27-29]. This remarkable physiological movement of the equine tooth requires a corresponding high rate of periodontal tissue remodeling [2]. It has already been shown that the equine PDL is characterized by a very high rate of cell proliferation and a distinct mode of collagen remodeling [30-32]. The utilization of the proposed high regenerative capacities of the equine PDL cells might offer promising new therapeutic approaches for treating typical equine disorders with high clinical relevance, i.e. treatment of destructive periodontal diseases, augmentation of the residual alveolar socket after tooth extraction and treatment of oromaxillary sinus fistula. Beyond the beneficial use for regenerative treatments in the fields of equine dentistry and equine craniofacial surgery, equine PDL cells might be also suitable for the regenerative treatment of disorders of other dense connective tissues of the equine body, in particular for the frequently injured digital flexor tendons.

The purpose of the presented study was to isolate, to culture and to characterize multipotent mesenchymal stromal cells (MSC) from the equine gingiva and from different PDL areas of the equine cheek tooth which measures up to 110 mm. The term multipotent mesenchymal stromal cells (MSC) is currently recommended by the International Society of Cellular Therapy (ISCT) in order to denominate fibroblast-like, plastic adherent cells with defined *in vitro *characteristics which have been previously termed mesenchymal stem cells [33].

## Results

### Isolation, growth and in vitro characterization of primary cells

**Figure 1:**
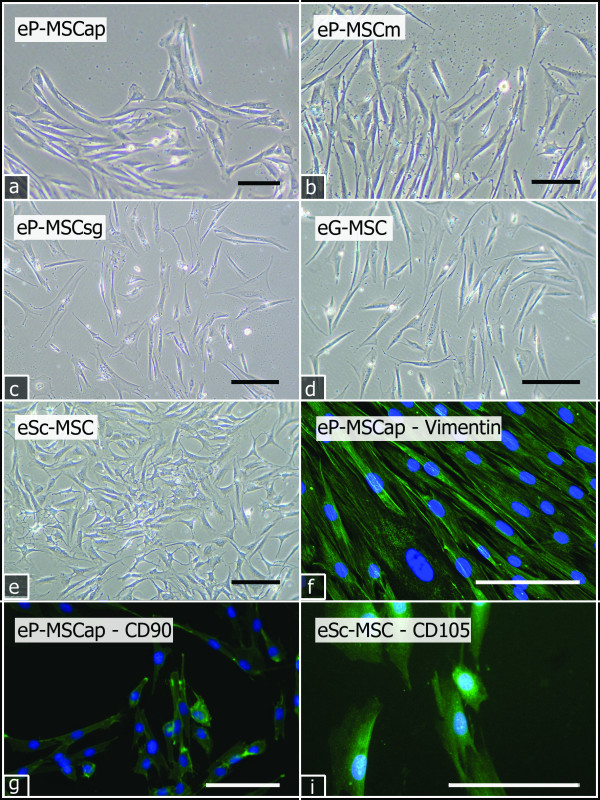
**Primary cells**. Reverse phase contrast (a-e) and fluorescent (f) images of cultured primary cells (P0). All primary cells showed fibroblastic morphology and adherence to plastic culture dishes. eP-MSCap (a): day 11; eP-MSCm (b), eP-MSCsg (c), eG-MSC (d): day 18; eSc-MSC (e), day 6. All cells were positive for vimentin (green), here exemplarily shown for eP-MSCap (f). Colony forming cells from passages 2 and 3 stained positive for CD90 (green), shown for eP-MSCap (g) and for CD105 (green), shown for eSc-MSC. Cell nuclei were stained with DAPI (blue). Scale bar = 100 μm.

The first plastic adherent cells were detected between two and three days after tissue prepara-tion. Primary cell cultures reached a confluence of 70%-90% at day 15 (eSc-MSC, range 11 to 18 days), day 25 (eP-MSC, range 15 to 39 days) or day 30 (eG-MSC, range 25 to 32 days). All cultures were proven to be enriched with fibroblasts by assessing cell morphology and immunostaining profiles. When examined with inverted phase contrast microscopy the primary cells exhibited long processes and displayed a spindle-shaped fibroblast-like morphology with few cells being binucleated (Figure 1a-e). All primary cultured cells stained positive for the intermediate filament vimentin (Figure 1f). None of the primary cultures contained pan-cytokeratin or CD31 positive cells, thereby proving the absence of epithelial and endothelial cells (data not shown). The selected cells could be cultured for more than five passages and maintained stable fibroblastic morphology and growth characteristics.

### Self-renewal capacity

#### CFU-F assays

**Figure 2:**
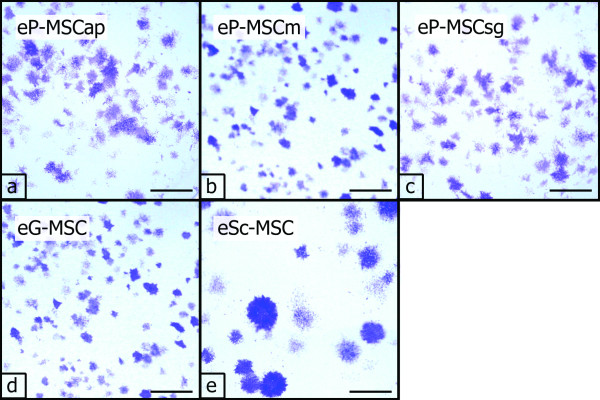
**CFU-F assays**. CFU-F assays, cell colonies (P2) were stained with 1% crystal-violet in me-thanol at day 15 of culture. eP-MSC (a-c) and eG-MSC (d) established multiple but small colonies, eSc-MSC (e) established fewer but larger colonies. Scale bar = 5 mm.

**Figure 3:**
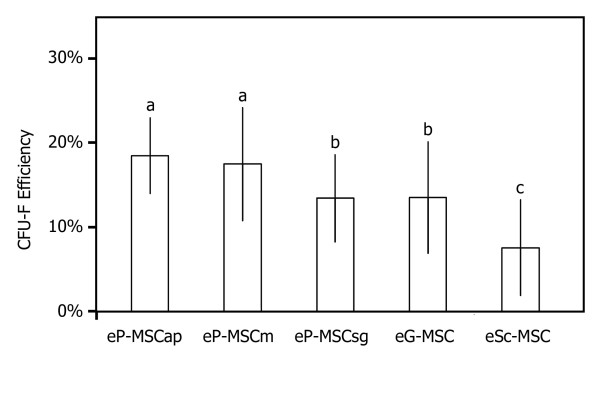
**CFU-F efficiency**. Efficiency of self renewal assessed by rate of colony formation in CFU-F assays. Columns illustrate mean CFU-F efficiency ± standard deviation. Six independent CFU-F assays were performed for each cell populations. Statistical data analysis: multiple variance analysis with repeated measurements; Tukey post-hoc-test for multiple mean value comparisons. P values < 0.05 were considered statistically significant. Significant differences among cell sources were denoted by a, b and c (all p-values < 0.019).

CFU-F assays demonstrated that all cultures contained a subpopulation of cells capable of generating new fibroblast colonies from single cells (Figure 2). Cells from the PDL (a-c) and from the eG-MSC (d) established multiple but small new colonies, whereas eSc-MSC established fewer but larger colonies. The calculated efficiency for CFU-F varied significantly between cell cultures obtained from different sources (Figure 3). eP-MSCap and eP-MSCm possessed the highest CFU-F efficiency, i.e. 18.45% (± 4.48%) and 17.45% (± 6.69%), respectively. eP-MSCsg and eG-MSC showed CFU-F efficiency of 13.43% (± 5.13%) and 13.50% (± 6.58%). eSc-MSC exhibited the lowest CFU-F efficiency of 7.59% (± 5.66%).

#### Population doubling time

**Figure 4:**
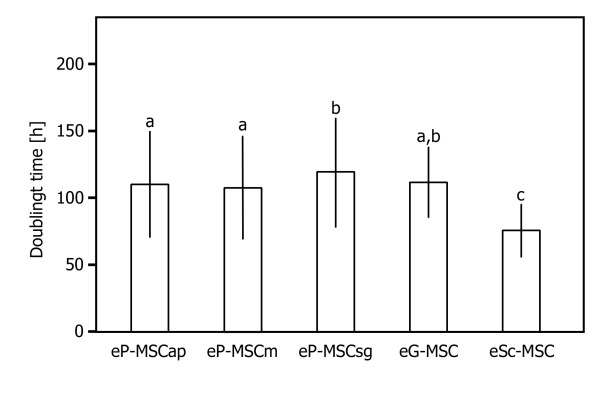
**Doubling time efficiency**. Efficiency for self renewal assessed by calculations of population doubling times. Columns illustrate mean doubling time ± standard deviation. Three independent doubling time assays were performed for each cell populations. Statistical data analysis: multiple variance analysis with repeated measurements; Tukey post-hoc-test for multiple mean value comparisons. P values < 0.05 were considered statistically significant. Significant differences among cell sources were denoted by a, b and c (all p-values < 0.031).

eSc-MSC showed significantly enhanced proliferation compared to all other cultured cell populations (p-values < 0.0021). Among the periodontal cells, eP-MSCap and eP-MSCm showed significantly higher growth rates (p-values < 0.0313) than eP-MSCsg (Figure 4).

#### Expression of stemness markers

Colony forming cells from all localizations (eSc-MSC, eP-MSCsg, eP-MSCm, eP-MSCap and eG-MSC) expressed the stemness markers CD90 and CD105. Immunocytochemical labeling demonstrated the presence of the membrane glycoprotein CD90 and the transmembrane glycoprotein CD105 predominantly on the cell surfaces.

### Multilineage differentiation assays

#### Osteogenic differentiation

**Figure 5:**
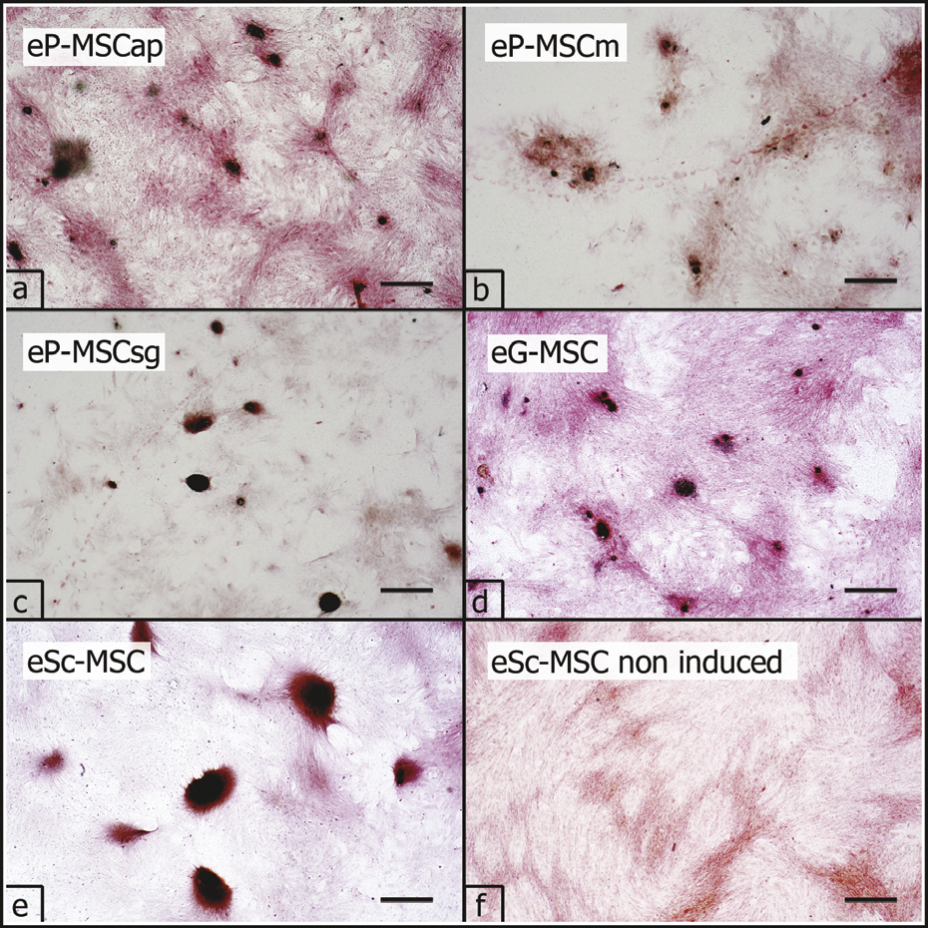
**Osteogenic differentiation**. Osteogenic differentiation (P3) as demonstrated by the presence of mineralized nodules stained black/purple with von Kossa staining (a-e). No mineralized nodules were apparent in non induced cell cultures, shown for eSc-MSC (f). Scale bar = 200 μm.

The osteogenic differentiation medium affected cell morphology and growth patterns. Almost all cultured cells changed their spindle-shaped fibroblast morphology and became stellate and irregular in shape. Instead of a confluent culture, cells formed multiple individual clusters with cells growing in several layers. All cell populations cultured in osteogenic differentiation medium produced a mineralized extracellular matrix stained positively with von Kossa. First mineralized nodules appeared at culture day 28 (Figure 5A-E). In controls cultures were kept in non-inductive culture medium, the cells preserved typical fibroblast morphology and growth characteristics with no formation of mineralized nodules in the extracellular matrix (Figure 5F).

#### Adipogenic differentiation

**Figure 6:**
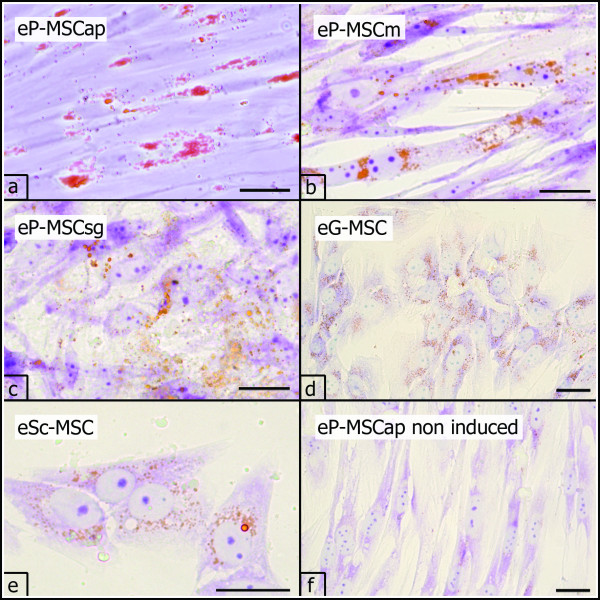
**Adipogenic differentiation**. Adipogenic differentiation (P3, P2) demonstrated by the accumulation of intracellular lipid droplets, stained orange with oil red O (a-e). No lipid droplets were present in non induced cell cultures, shown for eP-MSCap (f). Scale bar = 25 μm.

All cultures contained a subpopulation of cells capable of early stages of adipogenic differen-tiation. At day 23 of conventional adipogenic culture conditions (method 1) and at day three of adipogenic induction by 5% rabbit serum (method 2) these cells showed either a round and flat morphology or retained a spindle shaped phenotype. In all cases, adipogenic-induced cells contained single lipid droplets, stained orange by oil red O (Figure 6A-E). In none of the experiments did lipid droplets fuse to large vacuoles. Cells cultivated under non inductive conditions did not accumulate lipid droplets (Figure 6F).

#### Chondrogenic differentiation in three dimensional pellet cultures

**Figure 7:**
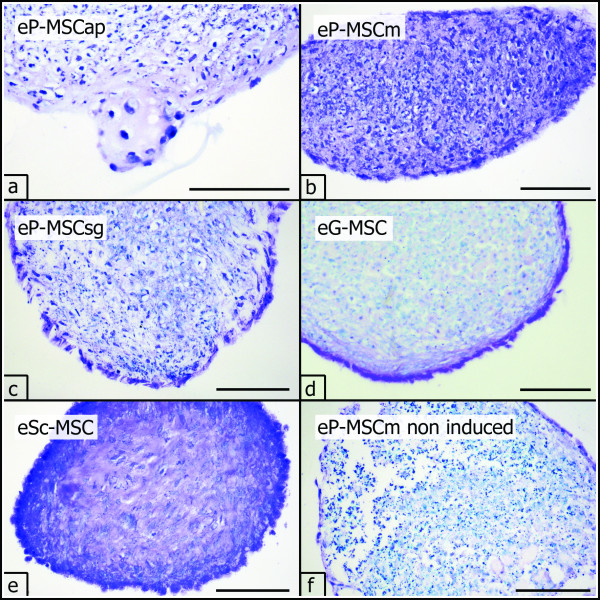
**Chondrogenic differentiation, toluidine blue staining**. Chondrogenic differentiation (P3) dem-onstrated in three dimensional pellet cultures. Sulphated proteoglycan deposition (purple metachromasia) in the extracellular matrix was assessed by toluidine blue staining (a-e). Only moderate cartilage matrix formation was present in non induced cell cultures, shown for eP-MSCm (f). Scale bar = 100 μm.

**Figure 8:**
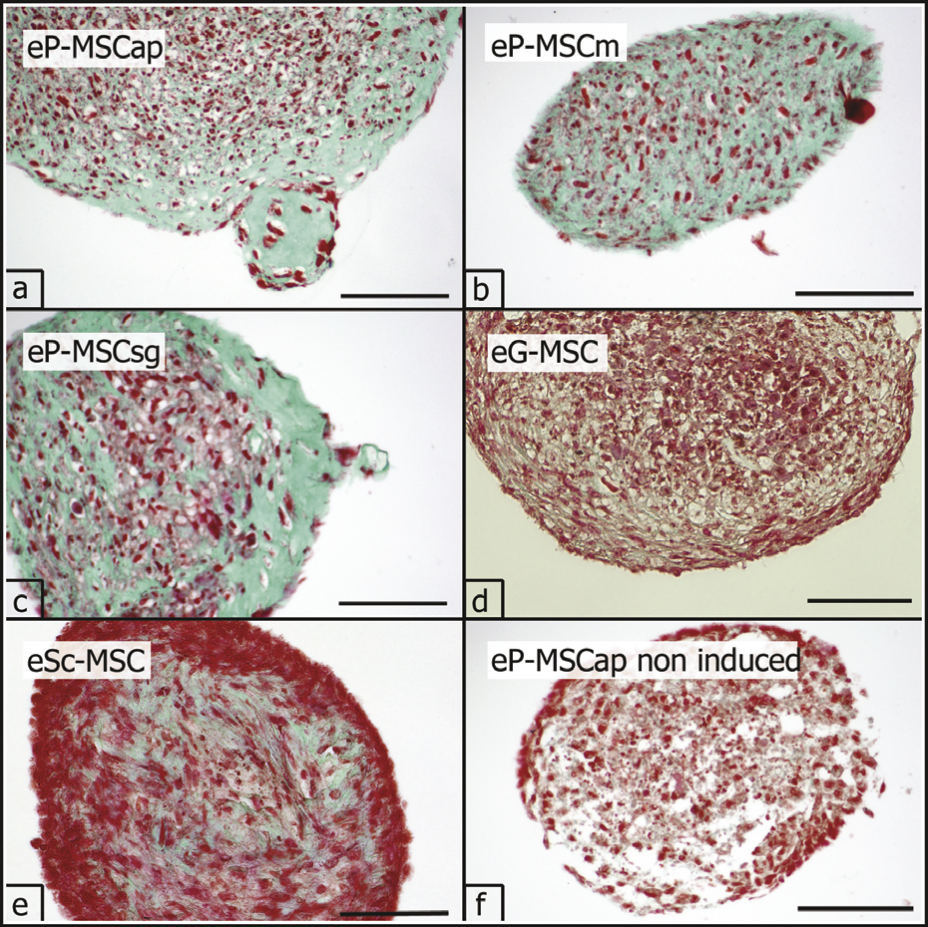
**Chondrogenic differentiation, Masson Trichrome staining**. Chondrogenic differentiation (P3) demonstrated in three dimensional pellet cultures. Collagen synthesis in the extracellular matrix (green) was confirmed with Masson Trichrome staining (a-e). Only moderate collagen formation was present in non induced cell cultures, shown for eP-MSCap (f). Scale bar = 100 μm.

Cultured cell pellets did not dissolve but maintained integrity and increased gradually in size during the 21-day culture period. Chondrogenic differentiation was assessed histologically by demonstrating the presence of cartilage-related matrix components in the specimens. Chondrogenic-induced pellets from all investigated cell sources showed intense purple metachromasia in toluidine blue staining, indicating a high content of sulfated proteoglycans (Figure 7A-E). Masson-Goldner-Trichrome staining revealed a high content of collagen fibers in chondrogenic-induced pellet cultures (Figure 8A-E).

In control pellets, cultivated in non-inductive medium, cells were assembled in loose ar-rangement with only few structural components in the extracellular matrix (Figure 7 and 8F).

#### RT-PCR

**Figure 9:**
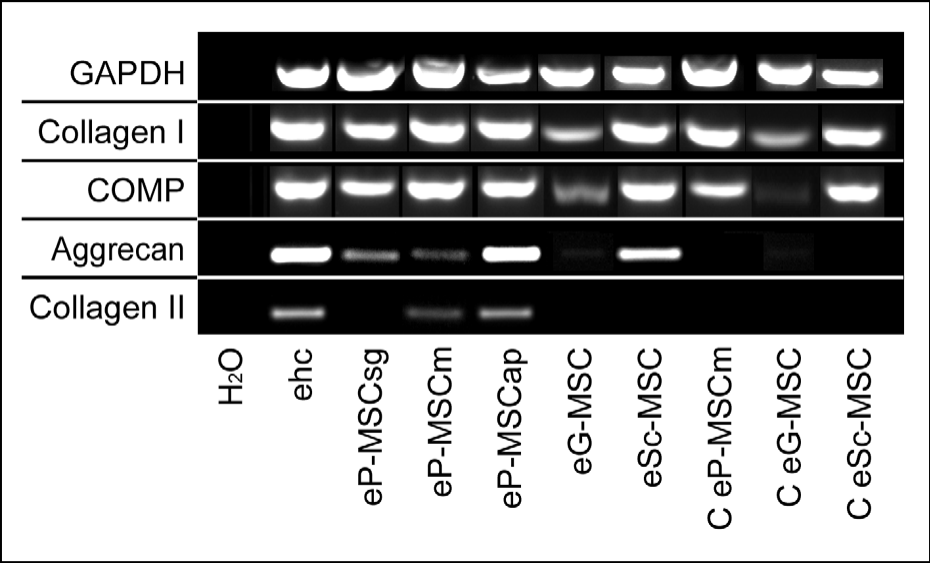
**RT-PCR**. RT-PCR analysis for indicated genes in pellet cultures (P3) after induction of chondrogenic differentiation. Cell pellets maintained in non inductive medium were used for controls (here shown for C eP-MSCm, C eG-MSC, C eSc-MSC). Extracted mRNA from equine hyaline cartilage (ehc) was used as a positive control. Note, only eP-MSCm and eP-MSCap show expression of mRNA for collagen type 2. Length of amplicons: GAPDH 341 bp, Collagen I 219 bp, COMP 238 bp, Aggrecan 147 bp, Collagen II 159 bp.

Further indicators of chondrogenic differentiation were assessed on mRNA level by RT-PCR. The expression of mRNA for GAPDH confirmed mRNA integrity and efficiency of reverse transcription. The predicted cDNA product of 341 bp was amplified from all investigated cell and tissue samples. The mRNA expression of collagen I and COMP were demonstrated in all pellet cultures, even those from control cultures kept in non-inductive medium (Figure 9). Collagen I mRNA was also expressed by non-induced cells cultured in two-dimensional cultures. Expression of mRNA for aggrecan was exclusively expressed by pellets cultured under inductive conditions. Amplicons for collagen II gene were expressed only in eP-MSCap and eP-MSCm (Figure 9).

## Discussion

### The equine periodontal ligament

The equine PDL simultaneously meets the opposing requirements of tooth support and conti-nuous tooth eruption at an exceptionally high rate under physiological conditions [25,31]. These distinct functions depend on dynamic properties which allow continuous periodontal remodeling in terms of renewal of dental cementum, periodontal ligament and alveolar bone [13,32,34]. Accordingly, periodontal remodeling is based on the presence of multiple cell types which are able to replenish the different tissues of the periodontium in a well orchestrated process [12,35,36].

It is widely considered that such complex processes as periodontal remodeling and functional regeneration depend on the presence of MSC within the PDL [18,32,37]. The presence of MSC has already been demonstrated in the PDL of men, e.g. [18-20], rats and sheep [14,21] but not in the PDL of horses. To the best of our knowledge this is the first study demonstrating the presence of MSC in the equine PDL.

Equine periodontal and subcutaneous MSC were identified according to distinct *in vitro *characteristics, i.e. plastic adherence, self-renewal capacity, expression of the stemness markers CD90 and CD105 and trilineage differentiation potency. Due to the fact that equine periodontal MSC have never been reported before, no comparative data exist. We therefore compared our results with non-equine periodontal MSC and also with non-periodontal equine MSC.

All harvested tissue samples contained plastic adherent cells which gave rise to primary cul-tures of fibroblast-like cells. These cells matched the typical in vitro appearance of fibroblasts from the PDL of non-equine species [38-40]. The presence of vimentin confirmed an ectomesenchymal origin of the cells and distinguished them from epithelial cells and endothelial cells.

In vitro self-renewal capacity of MSC is routinely demonstrated by CFU-F assays and doubling time experiments. Obtained results provide valuable information when considering prospective utilization of the investigated MSC for therapeutical use. The CFU-F efficiency is correlated with the quantity of MSC within their original in vivo tissue [41]. The in vitro doubling time provides quantitative information about the ability of the cells to expand in culture.

### CFU-F assays

Stro1+/CD146+ selected human periodontal MSC exhibited a CFU-F efficiency of 19.3% [42], which is in the same range as the values obtained for eP-MSCap (18.45%) and for eP-MSCm (17.45%). However, cells from other parts of the equine periodontium (eP-MSCsg and eG-MSC) possess lower CFU-F efficiencies of 13.43% and 13.50%. Given that the CFU-F efficiency represents an *in vitro *enumeration of a clonogenic subset of MSC *in vivo *as demonstrated by Kuznetsov et al. (2009) [41], the apical part of the equine PDL contains more MSC than other parts of the equine periodontium. The knowledge of site-specific quantities of MSC in the equine PDL might be of practical relevance regarding protocols for MSC isolation for further investigations. For non-equine species site-specific differences in the availability of PDL MSC have not been reported so far; and it is assumed that this issue remains important only for equine periodontal research due to the enormous length of equine teeth compared to other investigated species. However, even the highest CFU-F values of our cells were lower compared to CFU-F values obtained in equine bone marrow MSC, which show mean CFU-F rates of 27% [43].

### Population doubling time

Reported doubling times for equine MSC from different sources (bone marrow, adipose tissue) are in a range of 2.4 to 5 days [44,45]. These values are almost the same as demonstrated in equine periodontal MSC which double in number in 3.5 to 6 days. Cultured eP-MSCap and eP-MSCm proliferate at a significantly higher rate than eP-MSCsg. These in vitro findings are supplemented by corresponding in situ studies of the equine PDL. Warhonowicz et al. (2006) [31] demonstrated an elevated proliferation index in the apical level with decreasing proliferation indices towards the subgingival level. Such an asymmetric proliferation index with highest proliferative activity in the apical part of the PDL has been identified as playing a crucial role in continued tooth eruption [46,47].

### Trilineage differentiation

During osteogenic differentiation cells altered their shape and assembled in clusters. The cluster formation is considered a typical feature of osteogenic MSC differentiation in vitro [44]. Positive von Kossa staining confirmed the presence of calcium apatite in the extracellular matrix and thus also demonstrated successful osteogenic differentiation [48]. In addition previous studies showed that non-periodontal equine MSC formed visibly mineralized nodules within three weeks when cultured under osteogenic conditions [43,44,48]. In contrast our cells (ePDL-MSC, eG-MSC, eSc-MSC) did not show any presence of mineralized areas before day 28. This observation is in line with studies on non-equine periodontal MSC and suggests a suppressed capacity for extracellular matrix mineralization [20,48,49].

Equine periodontal and subcutaneous MSC showed early stages of adipogenic differentiation when cultured for 23 days in a conventional differentiation medium. The adipogenic differentiation was confirmed by the detection of small intracellular lipid droplets (oil red O staining). Yet, a final differentiation into mature adipocytes containing large, fused lipid vacuoles was not achieved. This is in line with the findings of other authors who have reported that equine MSC did not differentiate into mature adipocytes [50]. In a second experimental setting adipogenic differentiation was induced by incubation in a differentiation medium containing rabbit serum (5%) but no additional growth factors (i.e. dexamethasone, indomethacine, 3-isobutylmethylxanthine, insulin). This method had been proven to induce adipogenic differentiation in non-periodontal equine MSC at an optimal rate with minimized detachment of cells [51]. By using rabbit serum we observed accelerated adipogenesis (only three days culture time in induction medium) as also reported by recent investigations [44,51,52]. However, also in these experiments a terminal adipogenic differentiation with cells containing large, fused lipid vacuoles has not been observed. Specific investigations addressing the adipogenic differentiation capacity of adipose derived MSC assessed the expression of peroxisome proliferator activated receptor γ2 (PPARγ2) which plays an essential role in adipogenesis and has been widely accepted as a marker for terminal adipogenic differentiation [53,54]. Interestingly, even those equine adipose derived MSC did not display terminal adipogenic differentiation *in vitro *[50]. Nevertheless, although terminal adipogenic differentiation seems to be hampered in currently used *in vitro *culture systems for equine MSC, the demonstrated early adipogenic differentiation stage has been generally accepted to test adipogenic differentiation capacity [43,50,55].

Collagens and sulphated proteoglycans are characteristic constituents of the extracellular matrix in cartilage. Their deposition in chondrogenic-induced pellet cultures can be easily demonstrated by Masson-Goldner-Trichrome and toluidine blue staining [51]. However, staining intensity is influenced by several factors and obtained data should be confirmed by determining marker mRNAs for chondrogenic differentiation [44]. We therefore conducted RT-PCR experiments and demonstrated the expression of mRNA for collagen I, COMP and aggrecan in all chondrogenic-induced cell cultures. Interestingly, only eP-MSCap and eP-MSCm expressed transcripts for collagen II. According to the current concept of in vitro chondrogenesis of MSC, collagen I and COMP become upregulated in an early stage of chondrogenic differentiation. The expression of mRNA for aggrecan represents an intermediate stage and collagen II mRNA is expressed in a final stage [56]. Thus, only eP-MSCap and eP-MSCm passed all stages of in vitro chondrogenesis, while eP-MSCsg, eG-MSC and eSc-MSC displayed hampered chondrogenesis in vitro. Further investigations are needed to clarify whether these in vitro results reflect in vivo characteristics of the investigated MSC.

### Current therapeutic use of equine MSC

Recently, the use of so-called mesenchymal stem cells in equine medicine has gained a lot of scientific and commercial interest. However, applied cellular products have been defined according to different protocols and it is impossible to verify whether true MSC are used in different investigations [44,57].

Nevertheless, supposed MSC from different tissue sources (sternal bone marrow and adipose tissue) have been therapeutically used for regenerative therapies of typical equine musculoskeletal diseases, i.e. osteoarthritis [58-60] and core lesions in the superficial digital flexor tendon [61,62]. Cell-based regenerative therapy of equine tendinopathies turned out to improve clinical outcomes compared to conservative therapies [59,63,64]. Further, cell injections resulted in significantly improved tendons histologically [65,66]. At present, reported outcomes are still far from the biomechanical features of a healthy tendon and the currently used regenerative therapies need to be improved. The search for a tendon-like tissue containing available MSC populations has been identified as a promising approach in order to optimize cell-based therapy of equine tendon injuries [65]. This consideration is supported by the finding that MSC form different tissue sources possess different cellular properties due to the regulatory influence of their natural local microenvironment [67,68]. Significantly, tendon-derived MSC show a higher capacity for tenogenic differentiation when compared with bone marrow-derived MSC [49,69]. Unfortunately, the prevalence of MSC in tendons is very low and isolating suitable cell numbers appears to be impractical [69].

### Future prospects for the use of equine periodontal MSC

Considering a suggested tendon-like tissue source of MSC for equine tendon therapies, the obtained equine periodontal MSC might be promising candidates for such MSC. These isolated cells definitely possess typical MSC characteristics (plastic adherence, self-renewal capacity, and trilineage differentiation potency) and are obtained from a natural niche which greatly resembles tendon tissue. The particular in vivo function of periodontal MSC is reflected in their high in vitro expression of scleraxis, a tendon-specific transcription factor. Scleraxis expression is significantly higher in human periodontal ligament MSC when compared with human bone marrow derived MSC [20,70,71].

Equine periodontal MSC might also be a useful tool in order to develop successful therapies for equine periodontal disorders. Especially in aged horses periodontal diseases are a frequent problem with an incidence of up to 60 percent, often leading to tooth loss [72]. The search for predictable periodontal regeneration utilizing periodontal MSC has also attracted a lot of interest in the field of human periodontology and several promising therapeutical strategies have been proposed in the last years (for review see Huang et al. 2009 [73]).

Yet, a major problem of the use of equine periodontal MSC arises from their limited accessi-bility. Obtaining these cells for autologous applications can not be taken in to consideration. Hence, allogenic application techniques are required. Fortunately, recent investigations confirmed that MSC avoid or suppress immunological responses, usually causing rejection of allogeneic delivered cells [74,75]. Such remarkable immunomodulatory properties of MSC have also been explicitly demonstrated for human periodontal MSC [76].

Moreover, allogeneic application of equine MSC in diseased tendons was already been performed in experimental studies without causing immune response or tumor formation [62,77,78]. These clinical results have recently been supplemented by in vitro investigations which demonstrated the absence of MHC class II (a crucial immune activator) on equine MSC derived from bone marrow [57].

### Future prospects for classification and characterization of equine periodontal MSC

To provide an objective and comprehensive classification of the cells investigated, the rec-ommendations of the International Society for Cellular Therapies (ISCT) for the identification of non-human MSC [55] were applied. In our study the colony-forming cells showed the required characteristics, i.e. adherence to plastic culture dishes, and in vitro differentiation into osteoblastic, adipogenic and chondrogenic cells [55]. As those minimal criteria for the definition of non-human MSC were met by the isolated cells they were termed MSC.

For human MSC a third criterion is required, i.e. a well-defined profile of surface antigens [55,33]. A human MSC population should contain more than 95% of cells which express the surface makers CD73, CD90 and CD105, and less than 2% of the cells should express CD45, CD34, CD14 or CD11b, CD79α or CD19 and HLA class II [55,33,79]. Such a strict definition leads to a standardized and clear denomination of MSC and provides a substantial basis to compare results from experiments with MSC derived from different tissues of the body [55,79]. Only recently has the surface marker expression of human MSC from different dental tissues been thoroughly investigated and a useful panel of identifying marker molecules been recommended [73,80].

The difficulties associated with the establishment of uniform parameters for the characteriza-tion of putative MSC in human research are even more complicated in veterinary science. Unfortunately, the surface antigen expression of equine cells, in particular of equine MSC, must still be regarded as largely unknown [50,79,81]. Nevertheless, encouraging investigations showed reactivity of available antibodies against CD90 in equine bone marrow derived MSC [43], against CD 90 in equine adipose tissue derived MSC [44,50] and against CD105 in equine adipose tissue derived MSC [50]. Our results demonstrate that also MSC derived from other tissues of the equine body express CD90 and CD105 suggesting that these proteins might be used as universal markers for MSC in the horse. However, currently the panel of available equine stemness markers is very limited. The human MSC surface marker CD73 has been detected at an mRNA-level in equine MSC but not at a protein level so far [44]. Conflicting data exists for the expression of another putative MSC marker, CD13. This marker was recognized on equine MSC derived from peripheral blood but it was absent in MSC derived from adipose tissue [82,83]. Also demanded proof of the non-expression of particular CD antigens is still undetermined. Guest et al. (2008) [57] confirmed the non-expression of CD14 in equine MSC. However, others identified CD14 as an equine-specific characteristic of MSC [44]. Similar contradictory results have been reported for the expression [81] or non-expression [57] of the embryonic stem cell gene Oct4 in equine MSC. These inconsistent results emphasize the urgent need for future studies to identify and establish a useful and reliable panel of specific surface markers for equine MSC. Such surface markers would supplement and alleviate cell characterization and, even more importantly, would enable effective techniques for cell selection (immunomagnetic or fluorescence activated cell sorting). However, as long as an identifying antibody panel for equine MSC is not established, plastic adherence of colony-forming cells and trilineage differentiation capacity, should be regarded as minimal but adequate criteria for the identification of equine MSC [55,84].

## Conclusions

The presence of multipotent mesenchymal stromal cells within equine gingiva and periodontal ligament has been demonstrated. Protocols for cell isolation and cell expansion have been established. The obtained cell populations might be promising candidates for cell-based regenerative therapies in equine medicine, especially in the fields of craniofacial surgery, periodontal therapy and orthopedics. Further investigations are required to address the need for a panel of equine specific surface markers of MSC. In addition the cellular properties of equine periodontal MSC from different locations of the periodontium have to be compared with already characterized equine MSC from bone marrow and adipose tissue.

## Methods

### Animals

Samples were taken from four warm-blood horses (horse 1: 1-year-old, gelding; horse 2: 9-year-old, female; horse 3: 13-year-old, female, horse 4: 19-year-old, gelding). The animals had either previously been bought and then euthanized for the purpose of anatomical dissection courses, or had been euthanized for medical reasons at the Clinic for Horses of the University of Veterinary Medicine Hannover. The horses' ages, taken from the horses' passports, were verified by clinical examination of the dental status as recommended by Muylle (2005) [85]. All horses were free from dental or periodontal diseases.

Immediately after euthanasia, the lower jaw was removed and samples were taken from the PDL, the gingiva, and for control purposes also from the subcutis.

### Gingival samples

Samples were taken from the free gingiva at the buccal aspect of the cheek teeth region. After removing the gingival epithelium, tissue samples (sized approx. 10 mm × 4 mm × 2 mm) were obtained from the gingival lamina propria.

### PDL samples

Lower jaw segments were clamped in a bench vice, and the interdental spaces were widened to loosen the PDL of the mandibular cheek teeth. Subsequently, two fully erupted cheek teeth per horse were extracted. The intraalveolar parts of the teeth (termed reserve crown) measured up to 80 mm. PDL tissue samples were gently separated from the surface of the teeth at three horizontal levels (subgingival, mid-tooth, and apical).

### Subcutaneous samples

Samples were obtained from the subcutis of the masseteric region. After removing the skin, subcutaneous tissue samples (sized approx. 20 mm × 5 mm × 5 mm) were harvested.

### Primary cell culture

All tissue samples were placed in Dulbecco's phosphate buffered saline (DPBS) containing 100 U/ml penicillin, 100 μg/ml streptomycin, and 2.5 μg/ml amphotericin B (all constituents from PAA, Cölbe, Germany). Subsequently, samples were minced and washed three times in Dulbecco's modified Eagle's Medium (DMEM) containing 10% fetal bovine serum, 100 U/ml penicillin, 100 μg/ml streptomycin, and 2.5 μg/ml amphotericin B (all constituents from PAA). After a final wash, the tissue suspensions were centrifuged (500 g, 5 min), and the pellets incubated in DMEM containing 2% collagenase at 37°C for 15 min (collagenase II and collagenase IV from PAA). The dissolved tissues were centrifuged once again (500 g, 5 min) and then resuspended in standard culture medium (Dulbecco's modified Eagle's Me-dium supplemented with 1% minimum essential medium [non-essential amino acids], 10% fetal bovine serum, 0.02 mM/ml l-glutamine, 100 U/ml penicillin, 100 μg/ml streptomycin, 2.5 μg/ml amphotericin (all constituents from PAA). Finally, the cells were seeded into 6-well plastic culture dishes (Greiner Bio-One, Frickenhausen, Germany) and incubated in a humidified atmosphere (5% CO2, 37.0°C). Culture medium was changed after 48 h and thereafter every third day.

### Immunocytochemistry

Prior to the experiments, the purity of fibroblast cell cultures was confirmed by staining for vimentin and absence of staining for pan-cytokeratin and CD31 (anti-Vimentin, clone V9; anti-Pan-cytokeratin, clone KL1; anti-CD31; all antibodies mouse, monoclonal, DCS/BioGenex, Hamburg, Germany).

The expression of the stemness markers was assessed in cell cultures from passages 2 and 3 using anti-CD90 and anti-CD105 (both Antibodies, BD Bioscience, Heidelberg, Germany). For immunostaining, cells were rinsed with DPBS and fixed with methanol-acetone (1:1) at 4°C for 5 min. Afterwards non-specific binding was blocked by incubation with normal goat serum (DCS/BioGenex, Hamburg, Germany) for 30 min at room temperature. Subsequently, the specimens were incubated with the primary antibodies for 12 h at 4°C. Primary antibodies were used in the following dilutions: anti-Vimentin, anti-pan-cytokeratin 1:10, anti-CD31 1:30, anti-CD90 1:400, and anti-CD105 1:50. Then the probes were rinsed with DPBS and incubated with appropriate fluorochrome-conjugated secondary antibodies, i.e. goat anti-mouse (Alexa Fluor 594, Life Technologies GmbH, Darmstadt, Germany, dilution 1:1500 or FITC, Dianova, Hamburg, Germany, dilution 1:200) and goat anti-rabbit (Alexa Fluor 488, Life Technologies GmbH, Darmstadt, Germany, dilution 1:1500) for 45 min at room temperature. Cell nuclei were counterstained with DAPI or propidium iodide (PI). The nuclear staining agents were contained in mounting medium (Immunoselect Antifading Mounting Medium DAPI or Medium PI, Dianova, Hamburg, Germany). Immunoreactions were visualized with conventional fluorescence microscopy (Zeiss Axiovert 200 M, Carl Zeiss, Jena, Germany).

Controls for immunocytochemistry were prepared in three ways according to the recommen-dations of Burry (2000) [86]. Either the primary antibody was replaced by PBS, by secondary antibodies or sections were incubated with non-immune IgG (anti-rabbit IgG, Aldrich, Steinheim, Germany; anti-mouse IgG, Super Sensitive Control, DSC/Biogenex, Hamburg, Germany).

### Self-renewal capacity

#### Colony-forming unit-fibroblast (CFU-F) assays

To assess the capacity and efficiency for self renewal, cells (P2) were seeded at low density and new fibroblast colonies derived from single cells were counted. This procedure was referred to as colony-forming unit-fibroblast (CFU-F) assay. Following expansion cells were seeded in 6-well culture plates (50 cells/cm2). Day 15 cultures were fixed and stained with 1% crystal-violet in 100% methanol. Stained colo-nies made up of more than 20 cells were scored as CFU and were counted. Calculation of the CFU-F efficiency was performed according to the formula: CFU-F efficiency = (counted CFU-F/cells originally seeded) × 100. Routinely, six CFU-F assays were performed for each isolated cell population.

#### Population doubling time

Population doubling time was determined in 24-well culture plates at a density of 0.125 × 105 cells (P1) per well. After 24 h (t24h) non-adhesive cells in the medium were counted in every well and the adhesive cells (N0) were calculated. 24 h later (t48h) in three wells the adhesive cells were counted (N48h) and the doubling time (tD) was calculated according to the formula: tD = (log 2 × t)/(log N48h - log N0). The determination of cells in three wells was repeated eight times in 48 h-intervals with a change of medium on every third day. Routinely, population doubling time assays were performed in triplet for each isolated cell popu-lation.

### Statistical analysis

Recorded data were statistically analyzed using a multiple variance analysis with repeated measurements. Subsequently, a Tukey post-hoc-test for multiple mean value comparisons was performed to determine statistically significant differences. P values < 0.05 were considered statistically significant. Data analyses were conducted using SAS^® ^software Version 9.1 (SAS Institute, Cary, NC, USA).

### Multilineage differentiation

In order to control in vitro multilineage capacity differentiation experiments were conducted with cells from horse 1. Routinely, differentiation assays were performed in triplets for each isolated cell population.

#### Osteogenic differentiation

Cells (P3) were seeded in 24-well culture plates (0.2 × 105 cells/well). From the first day of incubation the cells were cultivated with osteogenic differentiation medium containing standard culture medium supplemented with 50 μg/ml l-ascorbic acid, 10 mM b-glycerophosphate, and 10 nM dexamethasone (all supplements from Sigma-Aldrich, Steinheim, Germany). The medium was changed every third day. 21, 28, and 35 day cultures were washed twice with DPBS and fixed in methanol-acetone (1:1) at 4°C for 10 min. Mineralization of the extracellular matrix served as an indicator of osteogenic differentiation. Mineralization was visualized using the von Kossa staining method [87].

#### Adipogenic differentiation

Cells (P3, P2) were plated in 24-well culture plates (0.2 × 105 cells per well) and grown to confluence in culture medium containing DMEM/HamsF12 (1:1, vol/vol), 20% fetal bovine serum, 100 U/ml penicillin and 100 μg/ml streptomycin. Subsequently, adipogenesis was induced by two different experimental methods.

##### Method 1

Cells were cultivated for three days in induction medium containing DMEM/HamsF12 (1:1, vol/vol) supplemented with 10% fetal bovine serum, 100 U/ml penicillin, 100 μg/ml streptomycin (supplements from PAA), 1 μM dexamethasone, 100 μM indomethacine, 500 μM 3-isobutylmethylxanthine, 700 nM insulin (supplements from Sigma-Aldrich). Finally, cultures were kept for one day in maintenance medium (DMEM/HamsF12 [1:1, vol/vol] supplemented with 10% fetal bovine serum, 100 U/ml penicillin, 100 μg/ml streptomycin, 700 nM insulin). This procedure was repeated four times and after the fourth cycle, cells were incubated for seven days in maintenance medium.

##### Method 2

Cells were cultivated for three days in culture medium (DMEM/HamsF12 [1:1, vol/vol], 100 U/ml penicillin and 100 μg/ml streptomycin) containing 5% rabbit serum (PAA, Cölbe, Germany).

Adipogenic differentiation was assessed by staining intracellular accumulated lipids with 0.5% oil red O (Sigma-Aldrich). To better distinguish the lipid droplets, cell cultures were counterstained with toluidine blue.

#### Chondrogenic differentiation

Chondrogenesis was induced in pellet cultures. Pellet cultures were prepared from 5 × 105 cells (P3) placed in 15 ml polypropylene tubes (Greiner Bio-One, Frickenhausen, Germany) and centrifuged at 500 g for 5 min at 10°C. Chondrogenic differentiation medium was prepared supplementing standard culture medium with 1% ITS+1, 10 ng/ml transforming growth factor 3 (TGF-3, Sigma-Aldrich), 8.8 μg/ml l-ascorbic acid and 0.1 μM dexamethasone (PAA). Pellet cultures were cultivated for 21 days with medium change every third day. At day 21 the cell pellets were fixed in 10% formalin for 24 h, and embedded in paraffin wax. Serial sections of the cell pellets were stained with Masson-Goldner-Trichrom and toluidine blue in order to demonstrate collagen content and sulfated proteoglycans within the extracellular matrix.

### RT-PCR

A set of chondrocyte-related genes (collagen I, COMP, collagen II, and aggrecan [88]) were assessed by RT-PCR. Total RNA and mRNA were isolated from chondrogenic induced pellet cultures, from non induced pellet cultures and from non-induced single layer cell cultures. Glyceraldehyde-3-phosphatedehydrogenase (GAPDH) mRNA was used as an internal control proving mRNA integrity and efficiency of reverse transcription. Tissue samples from equine hyaline cartilage (stifle joint), and superficial flexor tendon served as positive controls.

Total mRNA was isolated from tissues and cultured cells using the SV Total RNA Isolation System (Promega, Mannheim, Germany) according to the manufacturer's information. Complementary DNA (cDNA) was synthesized using SuperScript^® ^III reverse transcriptase (Invitrogen, Darmstadt, Germany). The amplification of cDNA was performed according to the manufacturer's recommendations using GoTaq^® ^DNA Polymerase (Promega, Mannheim, Germany) and master mix volumes of 20 μL containing 2 μL reverse transcript product.

#### Primer sets used and specific RT-PCR conditions were as follows

equine collagen 1 A2: 5' - TGGTGAAGATGGTCACCCTGGAAA - 3' and 5' - TCCTGCTTGACCTGGAGTTCCATT- 3' (XM_001492939), Annealing Temp.: 62.9°C, 35 cycles, amplicon 219 bp

equine COMP: 5'-AGTGTCGCAAGGATAACTGCGTGA-3' and 5'-TCCTGATCTGTGTCCTTCTGGTCA-3' (NM_001034034), Annealing Temp.: 61°C, 35 cycles; amplicon 238 bp

equine collagen 2A1: 5'-ATTCCTGGAGCCAAAGGATCTGCT-3' and 5'-TGAAGCCAGCAATACCAGGTTCAC-3' (NM_001081764), Annealing Temp.: 62.7°C, 35 cycles; amplicon 147 bp

equine aggrecan: 5'-TGGTGTCCTCTTCTTGTCGCTTTC-3' and 5'-ACGATACATTTGCTGTGCTTCGGC-3' (XM_001917528), Annealing Temp.: 62.7°C, 35 cycles; amplicon 159 bp

equine GAPDH: 5'-GGGTGGAGCCAAAAGGGTCATCAT-3' and 5'-AGCTTTCTCCAGGCGGCAGGTCAG-3' (XM_001488655), Annealing Temp.: 67°C, 35 cycles; am-plicon 341 bp

Amplified RT-PCR products were assessed by electrophoresis on a 2% agarose gel and visua-lized by ethidium bromide staining. A 100 bp DNA ladder served as molecular weight marker in each gel.

## List of abbreviations

MSC: Multipotent mesenchymal stromal cells; PDL: Periodontal ligament; CFU-F: Colony-forming unit fibroblast; eSc-MSC: equine subcutaneous multipotent mesenchymal stromal cells; eG-MSC: equine gingival multipotent mesenchymal stromal cells; eP-MSC: equine periodontal multipotent mesenchymal stromal cells; eP-MSCap: eP-MSC from the apical PDL; eP-MSCm: eP-MSC from the mid-tooth PDL; eP-MSCsg: eP-MSC from the subgingival PDL.

## Authors' contributions

NM designed the study, collected and processed the specimens, assembled and analyzed the data and helped with editing and revision of the manuscript. HG contributed to the study design, evaluated the data and obtained the funding. NH helped with the PCR, contributed to data analysis and interpretation. JDH helped with the PCR, contributed to data analysis and interpretation. CP contributed to data analysis and in-terpretation. CS contributed to the study design, helped with the collection and processing of the specimens, helped with the assembling and analysis of data, drafted and wrote the manuscript. All authors read and approved the final manuscript.
